# Supplementary material for: Long Non-Coding RNA GAS5 Promotes BAX Expression by Competing with microRNA-128-3p in Response to 5-Fluorouracil
Source: Biomedicines. 2022 Dec 26;11(1):58. doi: 10.3390/biomedicines11010058 (PMC9856034; doi:10.3390/biomedicines11010058)
Supplement: Supplementary file 1 [file biomedicines-11-00058-s001.zip › biomedicines-2068790-supplementary.pdf]

Supplementary Table S1. The sequences of oligos used in this study

Oligo	Primer	Sequence (5′ → 3′)	
		Forward	Reverse
qPCR	**GAS5**	CAGTGTGGCTCTGGATAGCA	TTAAGCTGGTCCAGGCAAGT
	**BAX**	TTTGCTTCAGGGTTTCATCC	ATCCTCTGCAGCTCCATGTT
	**GAPDH**	TGCACCACCAACTGCTTAGC	GGCATGGACTGTGGTCATGAG
Cloning	**pGAS5**	AAGGGGTACCTTTCGAGGTAGGAGTCGAC	AACCGCTCGAGGGATTGCAAAAATTTATTAAAATTG
	**BAX 3′UTR**	AAAAAGATCTTAAGATCAGAACCATCATGGGCT	AAAAGGTACCTTGTGTCCCGAAGGAGGTTT
	**MS2-GAS5**	AAAAGGTACCTTTCGAGGTAGGAGTC	AAAAGAATTCGGATTGCAAAAATTTA
PCR for BPD	**T7-GAS5**	CCAAGCTTCTAATACGACTCACTATAGGGAGATTTCGAGGTAGGAGT	GGATTGCAAAAATTTATTAAAATTG
